# Single-Cell and Multi-Omics-Based Characterization of Gastric Cancer Identifies TPP1 as a Potential Target for Gastric Cancer Progression and Treatment

**DOI:** 10.32604/or.2026.070208

**Published:** 2026-03-23

**Authors:** Yingying Zhao, Jiakang Ma, Rujin Huang, Shuxian Pan

**Affiliations:** 1 Department of Oncology, Yueyang People's Hospital of Hunan Normal University, Yueyang, China; 2 Department of Gastroenterology, First Affiliated Hospital of Zhengzhou University, Zhengzhou, China; 3 Henan Key Laboratory of Cancer Epigenetics, Cancer Institute, The First Affiliated Hospital, College of Clinical Medicine, Henan University of Science and Technology, Luoyang, China

**Keywords:** Gastric cancer, cancer-associated fibroblasts, single-cell RNA sequencing, tumor microenvironment, multi-omics analysis

## Abstract

**Background:**

Cancer-associated fibroblasts (CAFs) play critical roles in tumor progression and immunosuppression; however, their contribution to the functional classification and personalized treatment of gastric cancer remains poorly defined. This study aimed to identify effective therapeutic targets to facilitate individualized treatment strategies for patients with gastric cancer.

**Methods:**

Single-cell and bulk transcriptomic analyses were integrated to characterize gastric cancer fibroblasts. “*Seurat”*, “*Slingshot”*, and “*CellChat”* were used for dimensionality reduction, trajectory inference, and cell–cell communication analyses, respectively. Key metastasis-associated fibroblast modules were identified using High-dimensional weighted gene co-expression network analysis (hdWGCNA) to construct a prognostic model, which was further evaluated for immune infiltration, therapeutic response, and mutational features. The expression and function of the core gene tripeptidyl peptidase 1 (TPP1) were validated through immunoblotting, PCR, and functional assays.

**Results:**

Eight fibroblast subpopulations associated with gastric cancer metastasis exhibited distinct differentiation trajectories and transcriptional heterogeneity. Prognostic analysis indicated that metastasis-associated fibroblasts correlated with poor clinical outcomes. The high-risk subgroup showed marked immunosuppression, resistance to immunotherapy, and reduced mutational burden, with tumor progression–related pathways significantly enriched in this group. *In vitro* experiments further confirmed that TPP1 knockdown suppressed gastric cancer cell metastasis, invasion, and clonogenic capacity while inducing apoptosis.

**Conclusion:**

This study characterized the heterogeneity of gastric cancer–associated fibroblasts using single-cell transcriptomic analysis and established a prognostic model based on metastasis-related fibroblast markers. The model demonstrated strong predictive performance for patient prognosis, immune landscape, and immunotherapy response. Furthermore, the findings highlighted the pivotal role of TPP1 in gastric cancer progression and its potential as a therapeutic target.

## 1 Introduction

Gastric cancer remains among the leading causes of cancer-related death globally, characterized by significant heterogeneity affecting prognosis and treatment response [1–3]. Current therapeutic strategies primarily encompass surgical resection, perioperative chemoradiotherapy, and molecular-targeted therapies [4–6]. With advances in surgical techniques, precise tumor resection and lymph node dissection have become critical for improving the survival outcomes of patients with gastric cancer [7]. Surgical intervention remains contraindicated for patients presenting with metastatic disease [6]. Peritoneal dissemination is a prevalent metastatic pattern in gastric cancer, present in over 30% of patients at diagnosis [8,9].

Fibroblasts are pivotal regulators of tissue homeostasis and play essential roles in various physiological and pathological processes, including wound healing, inflammation, fibrosis, and tumor progression [10,11]. In the context of cancer, elucidating the phenotypic and functional heterogeneity of cancer-associated fibroblasts (CAFs) holds substantial prognostic and therapeutic value. Identifying CAF subpopulations selectively enriched in specific tumor subtypes may enable refined patient stratification and support the development of more precise and individualized treatment strategies [10,12]. CAFs represent a predominant component of the tumor stroma and are known to secrete a wide array of growth factors, pro-inflammatory cytokines, and extracellular matrix (ECM) components that collectively contribute to tumor cell proliferation, therapeutic resistance, and immune evasion [13].

Single-cell RNA-sequencing (scRNA-seq) is widely utilized to explore cellular heterogeneity and dynamically profile gene expression [14–16]. It effectively identifies cellular heterogeneity and accurately characterizes unique tissue cell populations [17–19]. Unlike bulk sequencing, single-cell sequencing enables precise analysis of intercellular communication, cellular functions, and differentiation trajectories within tissues [20]. In particular, it can accurately identify the dynamic evolution of the tumor immune microenvironment [21,22]. Growing evidence underscores the critical role of tumor-associated fibroblasts, whose transcriptionally and functionally distinct subpopulations exhibit diverse origins and developmental trajectories [21,23]. These subpopulations are closely linked to tumor-suppressive immune microenvironments, chemoresistance, immune evasion, tumor escape, and clinical treatment outcomes [21].

This study aims to characterize the heterogeneity of fibroblasts in gastric cancer using integrated single-cell transcriptomic and multi-omics data. By applying high-dimensional weighted gene co-expression network analysis (hdWGCNA), we identified metastasis-associated fibroblast (MAF) signatures and integrated them with differentially expressed genes to develop a robust prognostic model. This model effectively stratifies gastric cancer patients based on overall survival, immune landscape, response to immunotherapy, and genomic alterations. Notably, experimental validation identified TPP1 as a key MAF-related oncogene, driving gastric cancer progression. These findings offer new mechanistic insights into fibroblast-mediated metastasis and establish a foundation for personalized treatment strategies in gastric cancer.

## 2 Methods

### 2.1 Cohort Acquisition

Multi-omics datasets related to gastric cancer were retrieved from the Cancer Genome Atlas (TCGA; https://portal.gdc.cancer.gov/) and the Gene Expression Omnibus (GEO; https://www.ncbi.nlm.nih.gov/geo/) repositories. The cohorts analyzed included TCGA-Stomach Adenocarcinoma (STAD; n = 382), GSE62254 (Asian Cancer Research Group, ACRG; n = 300), GSE15459 (n = 200), GSE26901 (n = 109), GSE84433 (n = 357), and GSE84437 (n = 483). For TCGA, raw count data were used for differential expression analysis and subsequently converted to transcripts per million (TPM), followed by log2 transformation for immune-infiltration assessment and functional enrichment studies. All GEO datasets underwent standard normalization and stringent quality control before downstream processing. Additionally, the single-cell RNA-sequencing dataset GSE163558, generated from gastric cancer tissues, was obtained from GEO to construct the single-cell transcriptomic atlas.

### 2.2 Single-Cell Data Manipulation and Atlas Construction

**Figure A1:**
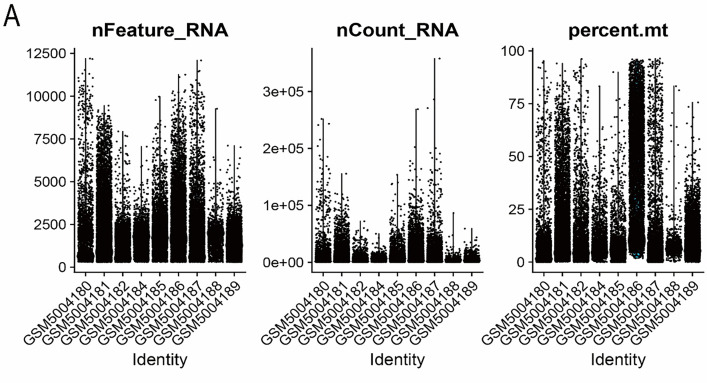

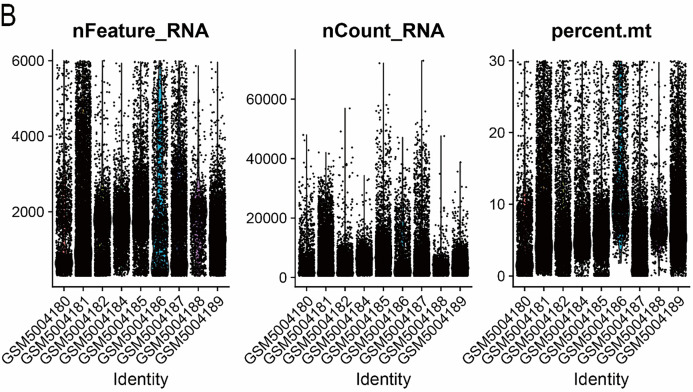
Visualization of single-cell data before and after quality control. (**A**) Before quality control. (**B**) After quality control.

Single-cell transcriptomic analyses were conducted using the “Seurat” package (version 4.4) [24]. After integrating the datasets, rigorous quality control (QC) was performed using predefined thresholds: 500–8000 detected genes per cell, 1000–60,000 total RNA counts, and mitochondrial transcript proportions below 30%. Cells meeting these criteria were retained for subsequent analyses. The filtered data were then normalized, batch effects were corrected, and highly variable genes were selected for downstream processing. The comparison of data characteristics before and after QC is presented in Fig. A1A,B. Cell clustering was conducted using the FindClusters function with a resolution of 0.4, which provided an optimal balance between cluster granularity and biological interpretability. Cell-type annotation and subsequent analyses were guided by canonical marker sets curated from publicly accessible databases and prior literature (CellMarker and CellMarker2.0; http://117.50.127.228/CellMarker/). Uniform Manifold Approximation and Projection (UMAP) embeddings were generated using the RunUMAP function in the “Seurat” package following principal component analysis (PCA). Cluster-enriched marker genes were identified using the *FindAllMarkers* function with the Wilcoxon rank-sum test as the statistical framework. Markers were retained only if they satisfied the following criteria: |log_2_FC| > 0.25, adjusted *p*-value < 0.05, and expression detected in at least 10% of cells within the corresponding cluster.

### 2.3 Cell Communication Analysis

Intercellular communication between major cell populations and fibroblast subpopulations in single-cell datasets was systematically analyzed using the “CellChat” package (Version 2.1.2) [25]. Intercellular communication probabilities were quantified using the computeCommunProb function, and pathway-specific communication intensities were assessed with computeCommunProbPathway. Aggregated intercellular networks were then constructed using the aggregateNet function. To facilitate comparative analyses between tumor and adjacent normal tissues, signaling pathway centrality metrics were calculated with netAnalysis_computeCentrality. Network-level alterations were visualized using interaction diagrams (netVisual_diffInteraction) and heatmaps (netVisual_heatmap), whereas significantly dysregulated signaling pathways were depicted through bubble plots (netVisual_bubble).

### 2.4 High-Dimensional Weighted Gene Co-Expression Network Analysis (hdWGCNA)

Identification of cellular module genes was performed using high-dimensional weighted gene co-expression network analysis (hdWGCNA) [26,27]. A soft-thresholding power of 12 was selected to generate a scale-free co-expression network and delineate module-specific gene sets. For each module, the top 20 hub genes were identified and visualized. UCell scoring was then employed to pinpoint modules most closely associated with metastasis-associated fibroblasts (MAFs), after which the corresponding module eigengenes were extracted for subsequent analyses.

***Single-cell tissue distribution analysis***

Single-cell tissue preference analysis is an advanced technique in single-cell RNA sequencing (scRNA-seq) that assesses the distributional bias of distinct cell types or subpopulations across different tissues or experimental conditions. Here, the STARTRAC algorithm [28] was employed to characterize the distribution patterns of fibroblast subtypes across primary gastric cancer samples and distinct gastric cancer subtypes.

### 2.5 Pseudotime Trajectory

Cell trajectory analysis was performed using the “Slingshot” package (2.14.0) to infer fibroblast differentiation trajectories [29]. Cell states and pseudotime distributions were visualized using plot_cell_trajectory from the “Monocle2” package (2.36.0) [30].

### 2.6 Prognostic Analysis of Metastasis-Associated Fibroblasts

Principal component analysis (PCA) was applied to the multi-omics gastric cancer cohorts using the metastasis-associated fibroblast signature genes to evaluate their contribution to interpatient heterogeneity. Patients were then classified into high- and low-score groups according to an optimal cutoff value derived from statistical modeling of the PCA scores. The prognostic relevance of these groups was examined using the “survival” package (version 3.8.3), and Kaplan–Meier curves were generated to visualize differences in overall survival. This framework provided an integrated evaluation of the clinical implications of fibroblast-related transcriptional programs in gastric cancer.

### 2.7 Fibroblast-Related Genes (FRGs) Prognostic Model Construction

To identify reliable prognostic biomarkers, a total of 731 candidate genes were compiled by integrating hdWGCNA-derived module genes, TCGA-derived differentially expressed genes, and markers associated with metastasis-related fibroblasts. Prognostic relevance was initially assessed using univariate Cox regression with a significance threshold of *p* < 0.05. Feature refinement was then performed using machine learning approaches, including Least Absolute Shrinkage and Selection Operator (LASSO) regression and random forest analysis, to pinpoint the most informative predictors. Based on these core genes, a prognostic risk-scoring signature was developed and subsequently validated in both the TCGA-STAD and ACRG (GSE62254) cohorts, demonstrating strong predictive performance and clinical utility.

### 2.8 Gene Ontology (GO) and KEGG Pathway Enrichment Analyses

Over-representation analysis (ORA) of differentially expressed genes (DEGs) from the training cohort was conducted using the clusterProfiler package (version 4.13.4). Gene symbols were converted to Entrez identifiers via org.Hs.eg.db (version 3.20.0), and all QC-filtered expressed genes were used as the reference background. Gene Ontology (GO) enrichment—covering Biological Process, Molecular Function, and Cellular Component categories—was performed with the enrichGO function, while KEGG pathway enrichment was assessed using enrichKEGG. Multiple hypothesis testing was addressed using the Benjamini–Hochberg correction (pAdjustMethod = “BH”), and significant terms were defined as those with an adjusted *p* value < 0.05 (with default gene set size filters of minGSSize = 10, maxGSSize = 500). When applicable, preranked gene set enrichment analysis (GSEA) was carried out with clusterProfiler, using gene lists ordered by signed log2 fold change, and reporting normalized enrichment scores (NES) along with FDR-adjusted *p* values. Enrichment results were visualized using the enrichplot package (version 1.26.2) and ggplot2 (version 4.0.0).

### 2.9 Analysis of Model-Related Biological Processes

Gene Set Variation Analysis (GSVA) and Gene Set Enrichment Analysis (GSEA) were employed to identify pathway-level differences between high- and low-risk groups, utilizing hallmark gene sets from the Molecular Signatures Database (MSigDB; https://www.gsea-msigdb.org/gsea/msigdb) and the “limma” package (version 3.62.1) for differential activity analysis [31]. To further elucidate the functional distinctions and underlying biological mechanisms associated with risk stratification, both GSEA and Gene Ontology (GO) enrichment analyses were performed, providing insights into the molecular pathways driving prognostic divergence.

### 2.10 Analysis of Model-Related Immune Infiltration and Immunotherapy

Tumor immune infiltration was comprehensively analyzed using multiple computational algorithms, including CIBERSORT [32], MCP-counter [33], xCell [34], EPIC, and quanTIseq [35], to characterize and compare the immune cell composition between high- and low-risk groups as defined by the prognostic model. The immune landscape was visualized using the “ComplexHeatmap” package (2.21.1) to facilitate the interpretation of intergroup differences [36]. In addition, tumor microenvironment scores (TMEscore) were computed using the “tmescore” package (0.1.4) to further assess the immunological context and its association with risk stratification [37].

### 2.11 Landscape of Mutation

Somatic mutation data for the TCGA-STAD cohort were obtained using the “UCSCXenaTools” package (1.6.0). Comprehensive analysis of the mutational landscape was performed with the “maftools” package (2.18.0) to characterize genomic alterations and their distribution across groups [38] and the mutational profiles were visualized using the Oncoplot function.

### 2.12 Boruta Feature Selection

To determine the most informative predictors among the fibroblast-associated candidate genes, wrapper-based feature selection was performed using the Boruta algorithm (R package Boruta; R v4.4.0). Boruta, which is built upon the random forest framework, evaluates the importance of each actual variable by comparing it against “shadow” features created through permutation of the original predictors. This iterative procedure yields statistically supported classifications—Confirmed, Tentative, or Rejected—for each gene.

### 2.13 Cell Culture and Transfection

Here, we use a cell line commonly used in gastric cancer research. Human gastric cancer cell lines (MKN45, HGC27, MGC803) and the normal gastric epithelial cell line (GES-1) were purchased from the Cell Bank of Shanghai Institute of Biochemistry and Cell Biology (Shanghai, China) in 2017 [39].

All cell lines were cultured in RPMI-1640 medium (Invitrogen, 11875093, Carlsbad, CA, USA) supplemented with 10% fetal bovine serum (FBS) and 1% penicillin–streptomycin under standard conditions. Gene silencing was performed using small interfering RNA (siRNA; GeneChem, Shanghai, China) delivered with Lipofectamine™ 3000 (Invitrogen, L3000015, USA). Prior to transfection, Lipofectamine™ 3000, siRNA, and Opti-MEM serum-free medium (Invitrogen, 31985070, USA) were combined and incubated at room temperature for 10 min according to the manufacturer’s instructions. All subsequent transfection steps were executed strictly following the reagent protocols. Total RNA was collected 48 h after transfection, and qRT-PCR was conducted to assess knockdown efficiency. All cell lines underwent short tandem repeat (STR) authentication and were confirmed to be free of mycoplasma contamination. Detailed mycoplasma test results are provided in the Appendix B. STR profiles of the gastric cancer cell lines matched their respective reference genotypes, with consistent allele numbers and complete concordance.

### 2.14 Human Tissues

Four paired human gastric cancer tissues and corresponding adjacent normal gastric tissues were obtained from the Department of Pathology at the First Affiliated Hospital of Zhengzhou University. All patients were histopathologically confirmed to have gastric cancer and had not received chemotherapy or immunotherapy prior to surgical resection. The study protocol was reviewed and approved by the Ethics Committee of the First Affiliated Hospital of Zhengzhou University (Approved No. 2024-KY-2227-002). Written informed consent was obtained from all participants in accordance with institutional guidelines and the ethical principles outlined in the Declaration of Helsinki. Immunohistochemical data from human tissue samples were obtained from The Human Protein Atlas (HPA: https://www.proteinatlas.org/).

### 2.15 Total RNA Extraction and Polymerase Chain Analysis

Total RNA was isolated from gastric cancer cell lines (MKN45, HGC27, and MGC803) and frozen gastric cancer tissues using TRIzol reagent (Invitrogen, 15596018CN, Carlsbad, CA, USA) following the manufacturer’s instructions. Complementary DNA (cDNA) was synthesized with the PrimeScript™ RT Kit (Takara, RR037A, Beijing, China), and RNA purity was assessed to exclude protein contamination. Quantitative reverse transcription PCR (qRT-PCR) was subsequently performed using SYBR^®^ Green Master Mix (Takara, RR820A, Beijing, China) under conditions specified by the manufacturer. Relative transcript levels were calculated using the 2^–ΔΔCt^ method. The primer sequences used were as follows:

GAPDH: Forward, GGTTGTCTCCTGCAGCTTCA; Reverse, TGGTCCAGGGTTTCTTACTCC. Tripeptidyl peptidase 1 (TPP1): Forward, GCAACTTTGCACATCAGGCA; Reverse, CCATGGAATGGGCACTCTGT.

### 2.16 Cell Counting Kit-8 (CCK-8) Assay

MKN45 and HGC27 cells, including untreated controls and siRNA-transfected groups, were seeded into 96-well plates at a density of 3000 cells per well. Cell viability was evaluated at 0 and 48 h using the Cell Counting Kit-8 (CCK-8; Beyotime, C0038, Shanghai, China) following the manufacturer’s protocol, and absorbance was recorded at 450 nm with a Flexa-200 microplate reader (Aosheng, Hangzhou, China). Each condition was assessed in triplicate, and the entire experiment was independently repeated three times to ensure technical and biological reproducibility.

### 2.17 Colony Formation

MKN45 and HGC27 cells, including control and siRNA-transfected groups, were seeded into 6-well plates at a density of 1000 cells per well. The culture medium was replaced every three days, and cells were maintained for 14 days to permit colony formation. Colonies consisting of more than 50 cells were considered valid, as assessed under a microscope (ChongGuang COIC XDS-1B; Chongqing, China). After removing the medium, wells were washed with balanced salt solution (pH 7.4, 0.01 M), fixed with 4% paraformaldehyde for 15 min, and stained with 0.1% crystal violet for 25 min. Colonies were photographed and quantified thereafter. Each condition was performed in triplicate, and the entire experiment was independently repeated three times to ensure reproducibility.

### 2.18 Transwell Assay

For the invasion assay, Matrigel (Corning, 354277, Corning, NY, USA) was evenly applied to the upper surface of Transwell inserts with 8 μm pore-sized membranes (Corning, 3428, New York, USA) and allowed to polymerize. MKN45 and HGC27 cells (2 × 10^5^ per insert) were seeded into the upper chambers in serum-free medium, while medium supplemented with chemoattractants was placed in the lower chambers. Cells were incubated for 24–48 h at 37°C. After incubation, non-invading cells on the upper membrane surface were gently removed. The inserts were then fixed in 4% paraformaldehyde for 15 min and stained with 0.1% crystal violet for 25 min. Invaded cells on the lower surface were imaged under a microscope, and five random fields per membrane were selected for quantification. Each condition was performed in triplicate, and the entire assay was independently repeated three times to ensure reproducibility.

### 2.19 Apoptosis Assay

MKN45 and HGC27 cells (2 × 10^5^ per well), including control and siRNA-transfected groups, were seeded into six-well plates and cultured for 24–48 h. After incubation, cells were collected and resuspended to obtain a single-cell suspension. A 50 µL aliquot of the suspension was stained with Annexin V and DAPI (Beyotime) following the manufacturer’s protocol. Staining was carried out at room temperature for 30 min in the dark. Apoptotic cell populations were subsequently quantified by flow cytometry using a BD Fortessa analyzer (Franklin Lakes, NJ, USA) to evaluate apoptosis induced by gene knockdown. The assay was independently repeated three times to ensure reproducibility.

### 2.20 Immunoblotting

Gastric cancer cell lines (GES-1, MKN45, HGC27, MGC803) and frozen Gastric cancer and normal tissues proteins were extracted using RIPA lysis (Beyotime, P0013B, Shanghai, China) buffer supplemented with protease and phosphatase inhibitors. Protein extraction was conducted under low-temperature conditions, followed by ultrasonic disruption and centrifugation. Protein concentrations were quantified using a BCA protein assay kit (Beyotime, P0010, Shanghai, China). The protein lysates were then mixed with 5× loading buffer at a ratio of 1:4 and denatured by boiling at 99°C for 10 min. For western blotting, 20 μg of total protein was loaded into each lane for SDS-PAGE and subsequent membrane transfer. After blocking at room temperature for 1 h, membranes were incubated overnight at 4°C with primary antibodies against TPP1 (1:3000, Proteintech, 25849-1-AP, Chicago, IL, USA) and GAPDH (1:3000, Proteintech, 10494-1-AP, Chicago, IL, USA). This was followed by incubation with HRP-conjugated secondary antibodies (1:20,000, Servicebio, GB25303, Wuhan, China) at room temperature for 2 h. Protein bands were visualized (ImageJ, 8.1 NIH, Bethesda, MD, USA) using enhanced chemiluminescence (ECL) (Servicebio, G2074, Wuhan, China) detection.

### 2.21 Statistical Analysis

Statistical analyses were performed using R software (version 4.4.0) and GraphPad Prism (University of California, San Diego, CA, USA; version 9.5). Data are presented as the mean ± standard deviation (SD). Group comparisons were conducted using two-tailed or one-tailed t-tests, or non-parametric tests, as appropriate based on data distribution. Univariate Cox proportional hazards regression was employed to identify prognostic factors, and Kaplan–Meier survival analyses were performed using the “survival” package in R (4.4.3) All bar graphs, scatter plots, and volcano plots were generated using the “*ggplot2”* package (4.0.0) in R, while forest plots were produced with the “*forestploter”* package (1.1.3). “ggalluvial” package (0.12.5) is used to draw Sankey diagrams. Venn diagrams generated with the “*VennDiagram”* R package (version 1.7.3). The “maftools” R package (version 2.18.0) was used to plot mutation waterfalls. A *p*-value < 0.05 was considered statistically significant. Statistical significance was indicated as follows: *p* < 0.05 (*), *p* < 0.01 (**), *p* < 0.001 (***), and *p* < 0.0001 (****).

## 3 Results

### 3.1 Analysis of Single-Cell Atlas of Gastric Cancer

**Figure 1:**
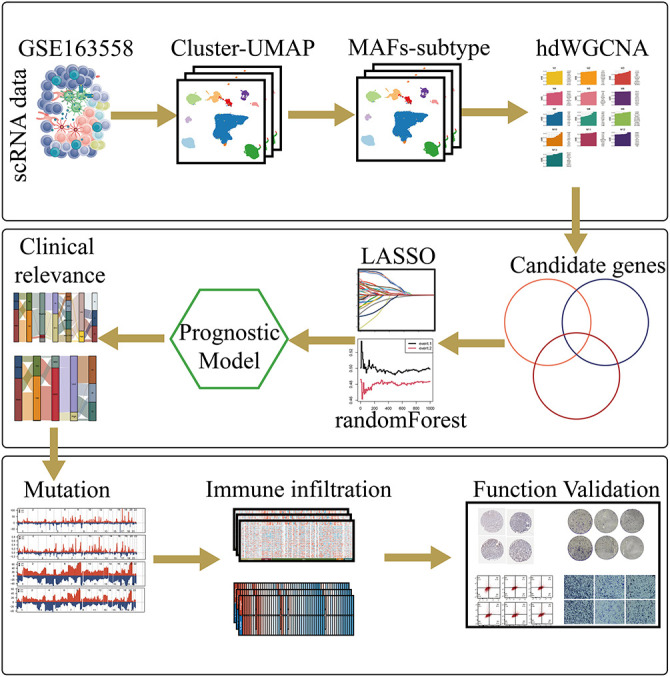
This schematic diagram provides an overview of the study’s integrated analytical framework. The workflow begins with data acquisition from public databases, followed by the integration of single-cell RNA sequencing and bulk multi-omics datasets. Metastasis-associated fibroblast subpopulations are then identified through clustering and functional annotation. Key gene modules are extracted using hdWGCNA, and a prognostic risk model is constructed and validated across independent cohorts. Finally, core genes—such as **TPP1**—are experimentally validated using *in vitro* assays to confirm their functional relevance in gastric cancer progression.

**Figure 2:**
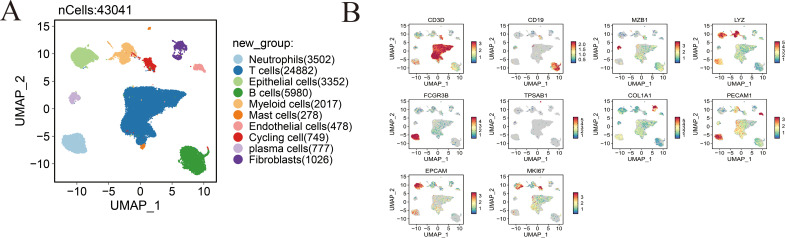

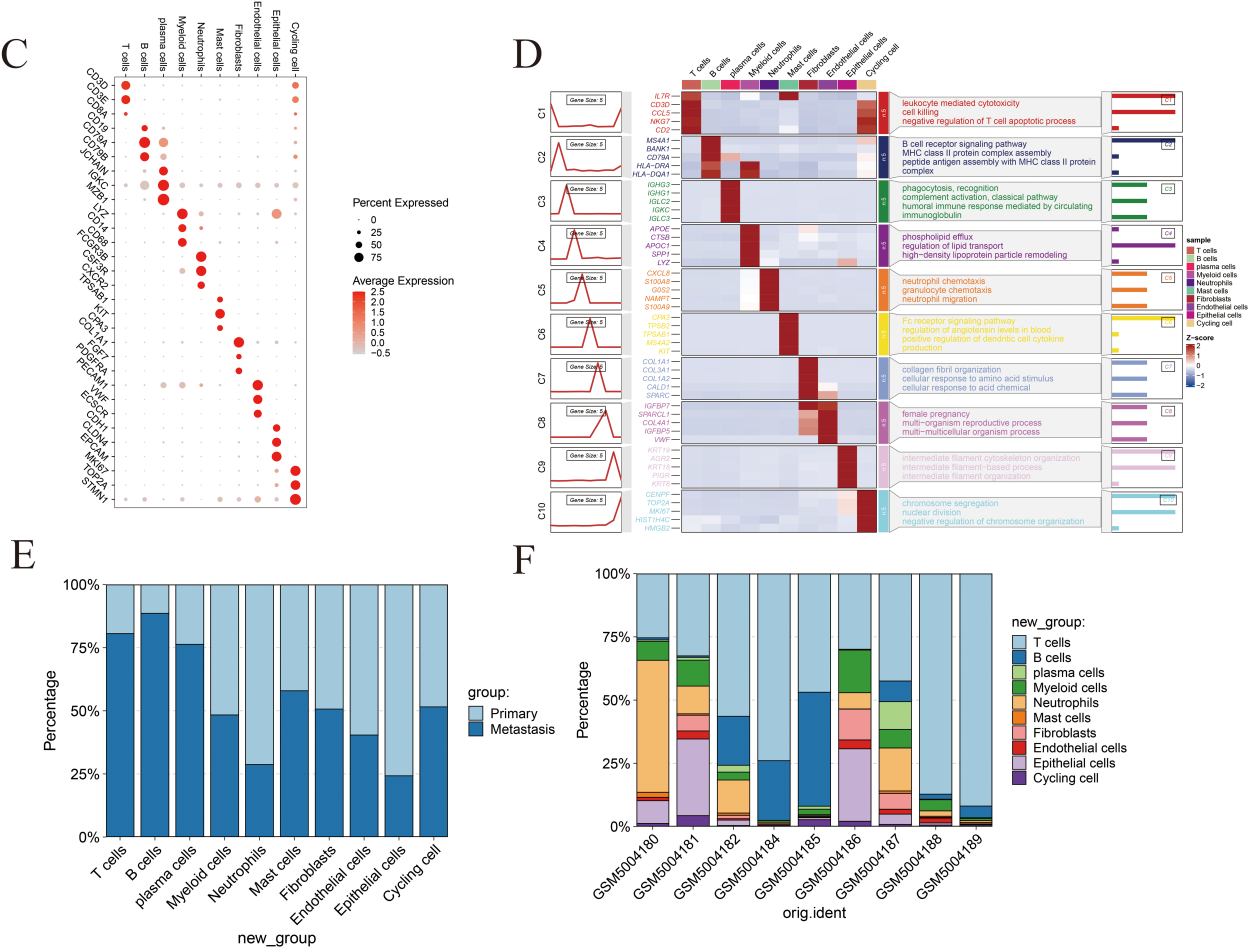
Comprehensive single-cell profiling of the gastric cancer microenvironment. (**A**) UMAP visualization of annotated cell populations, displaying distinct clustering of major cell types. (**B**) Expression levels of representative marker genes across defined cell populations, validating cell type annotations. (**C**) Bubble plot depicting the top five marker genes for each of the ten identified cell types; bubble size represents the proportion of expressing cells, and color intensity reflects average expression levels. (**D**) Heatmap showing the top five signature genes and their associated biological processes for each cell type, highlighting functional heterogeneity. (**E**) Bar graph illustrating the proportional distribution of various cell types across different tissue samples. (**F**) Infiltration abundance of the ten cell types across individual patient samples, demonstrating inter-sample variation in cellular composition.

Fig. 1 provides an overview of the analytical workflow and technical framework employed in this study. Following rigorous quality control and batch-effect correction of the gastric cancer single-cell RNA sequencing dataset, cell types were annotated based on canonical marker genes and reference databases, resulting in the identification of ten distinct cellular populations: neutrophils, T cells, endothelial cells, B cells, myeloid cells, mast cells, epithelial cells, cycling cells, plasma cells, and fibroblasts (Fig. 2A). The expression patterns of canonical marker genes corresponded closely with known cell identities, confirming the reliability of the annotation strategy (Fig. 2B). Representative marker genes for each population were visualized using a bubble plot (Fig. 2C), while a heatmap of the top five cell–type–specific marker genes per cluster further validated the classifications and highlighted the functional heterogeneity among cell types (Fig. 2D). Analysis of cell composition across individual patient samples revealed substantial interpatient variability, with T cells constituting the predominant immune population in most cases, underscoring the immune microenvironmental heterogeneity characteristic of gastric cancer (Fig. 2E,F).

### 3.2 Transcriptional and Functional Diversity of Fibroblasts in Gastric Cancer

**Figure 3:**
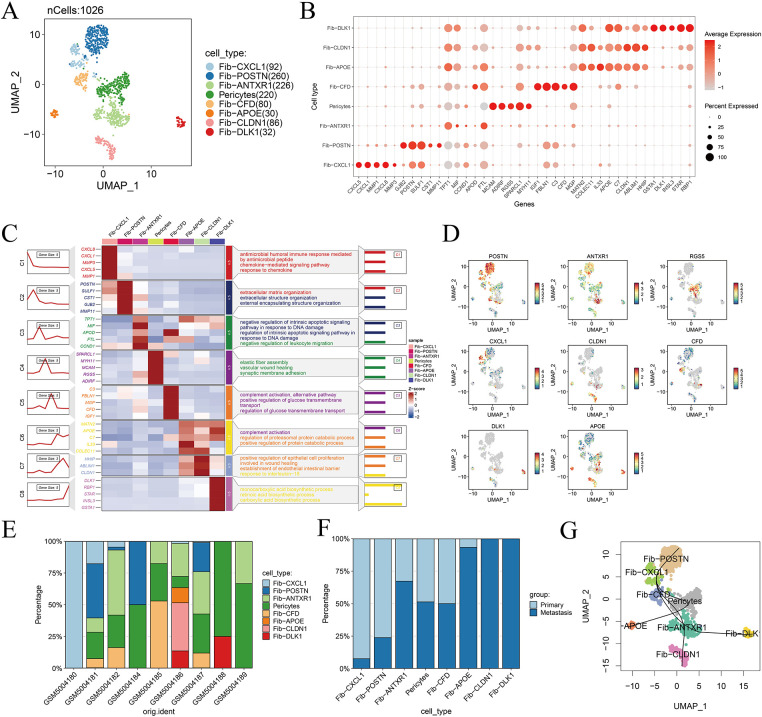
Dissecting fibroblast heterogeneity in the gastric cancer microenvironment. (**A**) UMAP visualization of fibroblast subpopulations, illustrating distinct clustering of eight fibroblast subtypes. (**B**) Bubble plot displaying the top five marker genes for each fibroblast subtype; bubble size represents the percentage of cells expressing the gene, and color indicates average expression level. (**C**) Heatmap of enriched biological processes associated with each fibroblast subtype, highlighting functional specialization. (**D**) Expression patterns of representative marker genes across the eight identified fibroblast subtypes. (**E**) Infiltration abundance of each fibroblast subtype across individual samples. (**F**) Distribution of fibroblast subtypes across different tissue types, demonstrating spatial heterogeneity. (**G**) Slingshot trajectory plot showing the inferred developmental progression and state transitions of fibroblast subtypes over pseudotime.

Eight distinct fibroblast subpopulations were identified, including Fib-CXCL1, Fib-POSTN, Fib-ANTXR1, pericytes, Fib-CFD, Fib-APOE, Fib-CLDN1, and Fib-DLK1 (Fig. 3A). Subtype-specific marker genes were visualized using a bubble plot to confirm annotation accuracy (Fig. 3B), while a heatmap of the top five differentially expressed genes per cluster highlighted the unique transcriptional programs and functional specialization of each fibroblast subset (Fig. 3C). UMAP visualization of representative marker gene expression demonstrated strong spatial concordance with defined cluster identities, further validating the robustness of subtype classification (Fig. 3D). Analysis of fibroblast subtype distribution across patient samples revealed marked interpatient heterogeneity, emphasizing the complexity and diversity of fibroblast populations within the gastric cancer microenvironment (Fig. 3E,F). Moreover, fibroblast lineage trajectories inferred using the Slingshot algorithm uncovered dynamic differentiation pathways and transitions among fibroblast subpopulations across temporal and cellular states (Fig. 3G).

### 3.3 Metastasis-Associated Fibroblast Subtypes Correlate with Adverse Clinical Outcomes

**Figure A2:**
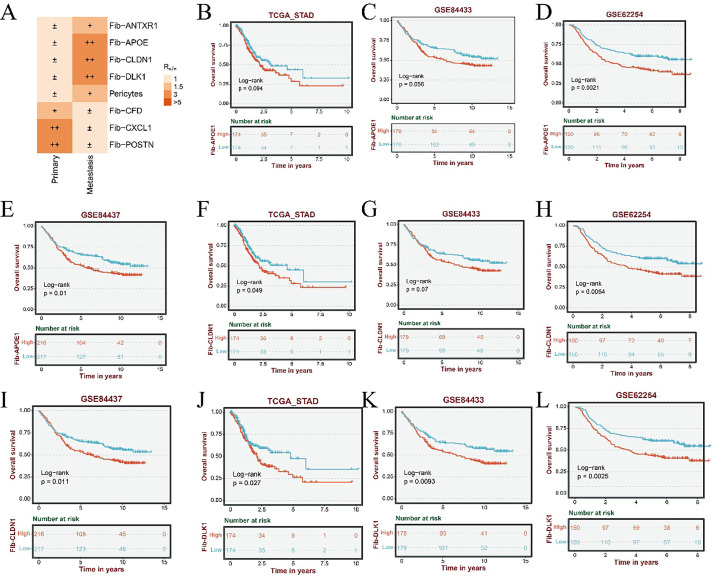

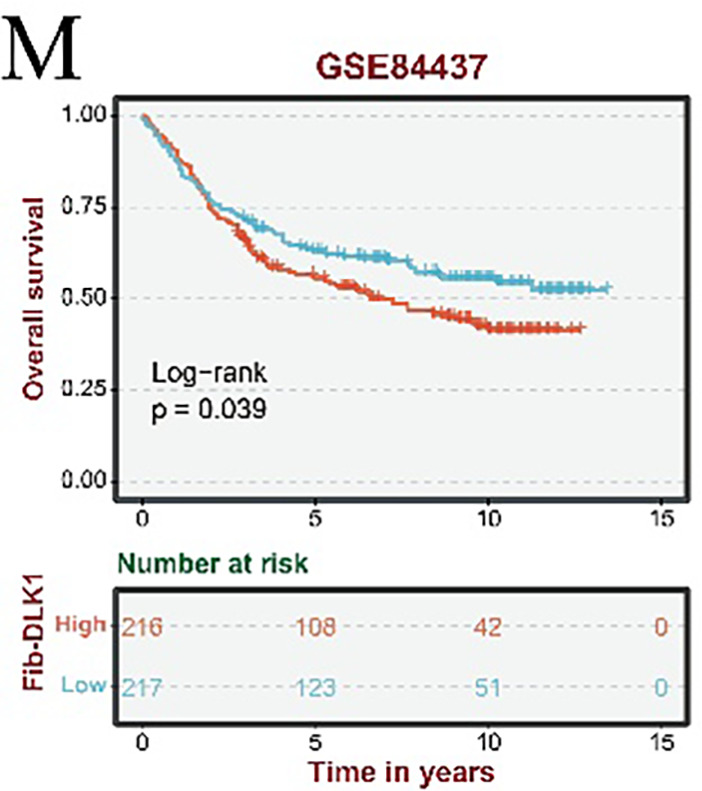
Subtype-specific distribution patterns and prognostic implications of gastric cancer fibroblasts. (**A**) Tissue preference analysis reveals enrichment patterns of fibroblast subtypes across metastatic and non-metastatic gastric cancer samples (± indicates low distribution degree; + indicates a moderate distribution; ++ represents a higher distribution). (**B**–**E**) Kaplan–Meier survival analyses of the **Fib-APOE** subtype in the TCGA (**B**), GSE84433 (**C**), GSE62254 (**D**), and GSE84437 (**E**) cohorts. (**F**–**I**) Prognostic evaluation of the **Fib-CLDN1** subtype in the TCGA (**F**), GSE84433 (**G**), GSE62254 (**H**), and GSE84437 (**I**) datasets. (**J**–**M**) Survival impact of the **Fib-DLK1** subtype assessed across the TCGA (**J**), GSE84433 (**K**), GSE62254 (**L**), and GSE84437 (**M**) cohorts.

Tissue preference analysis of fibroblast subpopulations revealed significant enrichment of Fib-APOE, Fib-CLDN1, and Fib-DLK1 subsets in metastatic gastric cancer samples, indicating a strong association with tumor metastasis (Fig. A2A). The top 50 marker genes for each fibroblast subtype were identified using the FindAllMarkers function, and principal component analysis (PCA) was performed on the TCGA cohort to derive subtype-specific signature scores. Kaplan–Meier survival analysis demonstrated that elevated scores for the three metastasis-associated fibroblast signatures were significantly correlated with reduced overall survival in patients with gastric cancer (Fig. A2B–M).

### 3.4 Cell Communication

**Figure A3:**
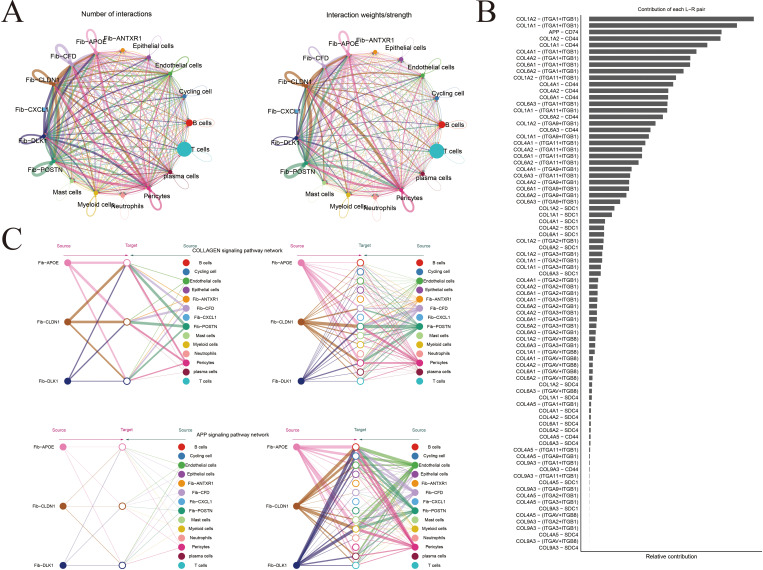

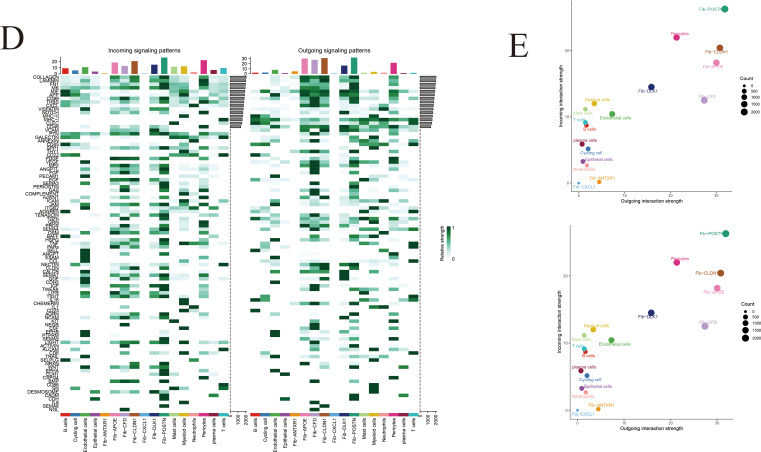
Inference of cell–cell communication networks at single-cell resolution. (**A**) Cell–cell communication network among major cell types in gastric cancer. Line thickness and color intensity represent the strength of inferred signaling interactions. (**B**) Bar plot illustrating the relative contribution of key signaling pathways to intercellular communication. (**C**) Hierarchical network diagram depicting the directional flow of cell–cell communication through the COLLAGEN and APP signaling pathways. (**D**) Heatmap showing the number and strength of pathway-mediated interactions between distinct cell populations. (**E**) Quantitative comparison of overall communication strength among different cell types within the tumor microenvironment.

Comprehensive network communication analysis uncovered intensive intercellular signaling among fibroblast subsets, with notably strong crosstalk between metastasis-associated fibroblasts (MAFs) and myeloid cells, underscoring a potential regulatory axis within the tumor microenvironment (Fig. A3A). The COL1A2 and APP pathways were identified as central mediators of fibroblast-driven communication, with MAFs engaging in pronounced bidirectional signaling with myeloid populations through these specific axes (Fig. A3B,C). Subsequent analysis revealed that the COL1A1 signaling pathway exhibited markedly elevated outward signaling specifically within MAF subsets, suggesting its involvement in tumor-promoting stromal remodeling (Fig. A3D). Moreover, signal strength profiling indicated that the Fib-CLDN1 and Fib-APOE subtypes displayed particularly high levels of intra-subtype communication, implicating their roles in reinforcing localized fibroblast–fibroblast interactions and maintaining niche-specific functional heterogeneity (Fig. A3E).

### 3.5 hdWGCNA Reveals Functional Gene Modules in Metastasis-Associated Fibroblasts

**Figure 4:**
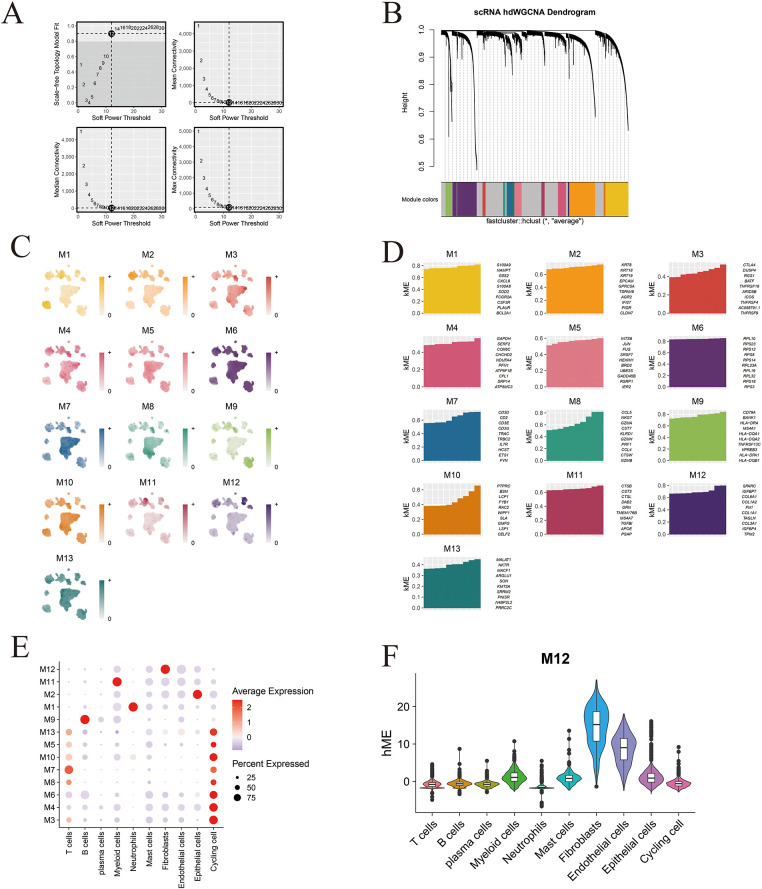
High-dimensional co-expression network analysis reveals MAF-related modules. (**A**) Schematic representation of the scale-free topology model fit across a range of soft-thresholding powers, used to determine the optimal value for network construction. (**B**) Hierarchical clustering dendrogram generated by hdWGCNA, identifying 13 distinct gene co-expression modules. (**C**) Module feature scores for the 13 gene modules, reflecting their relative contribution to overall variance. (**D**) Visualization of the top ten hub genes from each of the 13 identified hdWGCNA modules. (**E**) Bubble plot depicting the relationship between module scores and annotated cell types; bubble size indicates the proportion of cells, while color reflects module expression level. (**F**) Distribution of Module 12 (M12) scores across the ten major cell types, highlighting its specificity for fibroblast populations.

**Figure A4:**
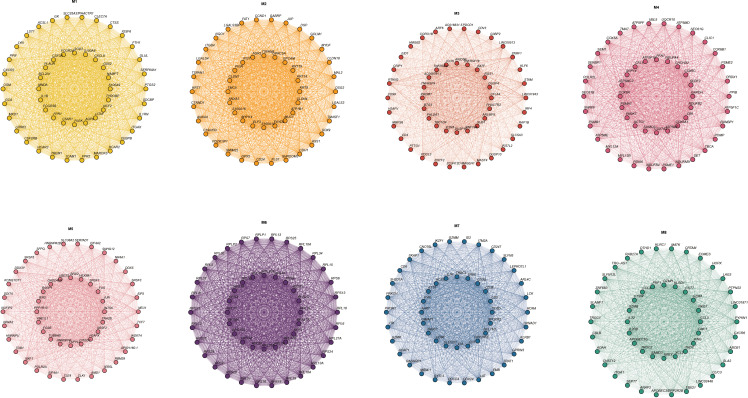

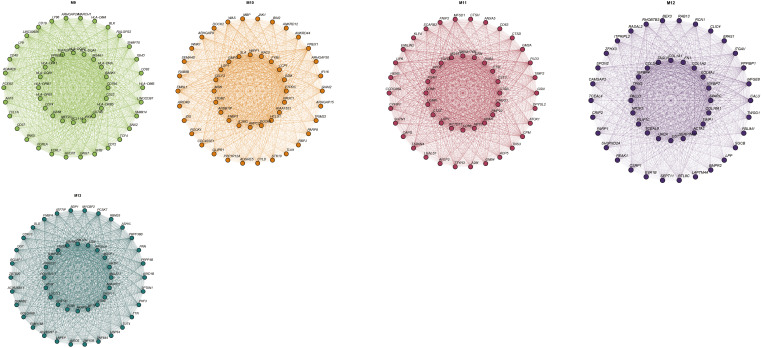
Top 20 genes in different modules of hdWGCNA.

A soft-thresholding power of 12 was applied to construct a scale-free gene co-expression network, resulting in the identification of 12 distinct and biologically robust gene modules (Fig. 4A,B). Module distribution across different cell populations was visualized using UMAP, revealing distinct enrichment patterns specific to individual cell subtypes (Fig. 4C). The ten most highly connected hub genes within each module are presented in Fig. 4D. Notably, bubble plot analysis demonstrated that Module 12 (M12) exhibited a strong and selective association with fibroblast populations (Fig. 4E). Fig. A4 further displays the top ten eigengenes per module, highlighting key contributors to module functionality. Among all modules, the M12 eigengene score was highest within fibroblast clusters, confirming its specificity and biological relevance to this stromal compartment (Fig. 4F).

### 3.6 Machine Learning–Driven Construction of a Prognostic Classifier

**Figure 5:**
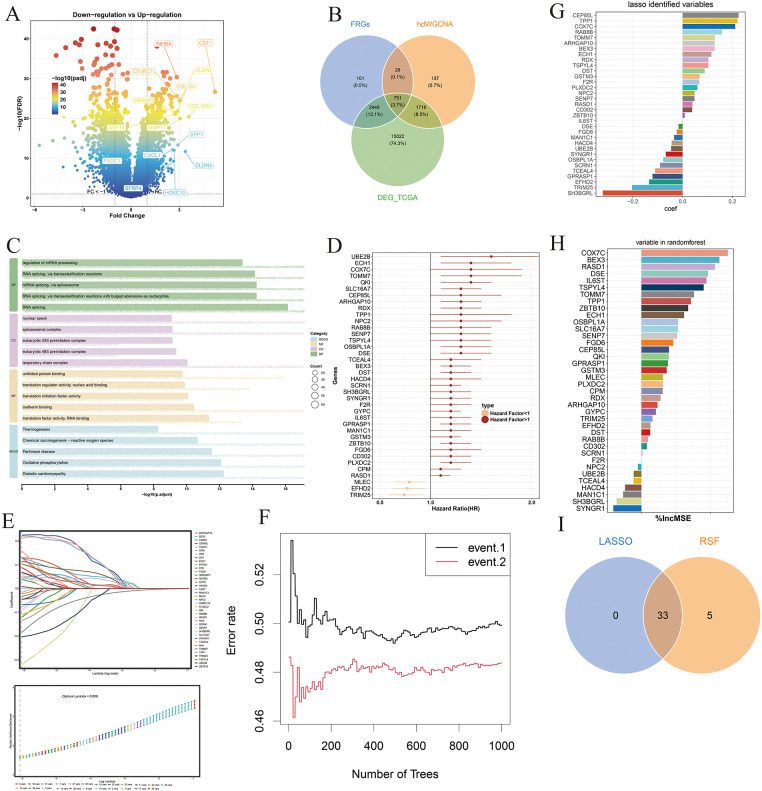
Development of a fibroblast-associated prognostic signature. (**A**) Volcano plot displaying differentially expressed genes (DEGs) in the TCGA-STAD cohort; red dots indicate statistically significant genes. (**B**) Venn diagram showing the intersection of the hdWGCNA module genes, metastasis-associated fibroblast-related genes (MFRGs), and TCGA-STAD DEGs, used to define the candidate gene set. (**C**) Functional enrichment analysis of the candidate genes reveals significantly involved biological processes and pathways. (**D**) Forest plot of prognostic genes identified through univariate Cox regression analysis. (**E**) LASSO regression applied to refine prognostic gene selection. (**F**) Random forest algorithm used to identify high-importance prognostic genes. (**G**) Bar graph illustrating non-zero coefficient genes selected by LASSO regression. (**H**) Bar chart highlighting the top-ranking variables by importance score in the random forest model. (**I**) Venn diagram showing the final intersection of LASSO and random forest results, defining the core prognostic gene set used for model construction.

Differentially expressed genes (DEGs) were first identified in the TCGA-STAD cohort (Fig. 5A). These DEGs were then intersected with gene sets derived from metastasis-associated fibroblasts (MAFs) and key modules identified through high-dimensional weighted gene co-expression network analysis (hdWGCNA), yielding a set of overlapping candidate genes (Fig. 5B). Functional enrichment analysis of these intersecting genes revealed significant involvement in biological processes and pathways related to RNA splicing, translation factor activity, RNA binding, and oxidative phosphorylation (Fig. 5C). To evaluate their prognostic relevance, univariate Cox regression analysis was performed on the overlapping gene set, and genes significantly associated with overall survival were retained for further analysis (Fig. 5D). These prognostically significant genes were subsequently subjected to Least Absolute Shrinkage and Selection Operator (LASSO) regression and random survival forest (RSF) modeling to refine the feature set (Fig. 5E,F). Genes with nonzero coefficients in the LASSO model and high importance rankings in the RSF model were identified (Fig. 5G,H), and the intersection of both methods yielded 33 robust feature genes (Fig. 5I), which were ultimately used to construct the prognostic risk model.

### 3.7 Downstream Pathway Analysis of Prognostic Gene Signatures

**Figure 6:**
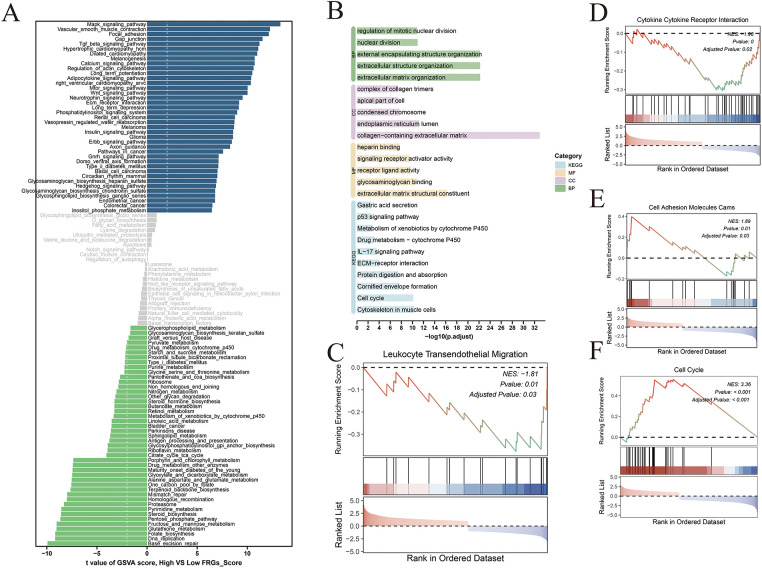
Comparative functional enrichment analysis of high- and low-risk groups. (**A**) Bar plot showing the results of Gene Set Variation Analysis (GSVA) comparing high- and low-risk groups; blue bars represent pathways enriched in the high-risk group, while green bars indicate enrichment in the low-risk group. (**B**) Gene Ontology (GO) and KEGG pathway enrichment analysis of differentially expressed genes between risk groups, highlighting distinct biological processes and signaling pathways. (**C**–**F**) Gene Set Enrichment Analysis (GSEA) illustrating significantly enriched pathways in the high- and low-risk groups, reflecting functional divergence associated with risk stratification.

Gene Set Variation Analysis (GSVA) revealed significant enrichment of oncogenic signaling pathways—including MAPK, TGF-β, and Wnt signaling—in the high-risk group, indicative of enhanced tumor proliferative potential (Fig. 6A). In contrast, the low-risk group exhibited preferential enrichment in pathways associated with genomic stability, whereas the high-risk group showed increased activation of p53, IL-17, and receptor–ligand signaling pathways, reflecting a more dysregulated immune milieu and heightened proliferative signaling (Fig. 6B). Consistently, Gene Set Enrichment Analysis (GSEA) demonstrated that the low-risk group was enriched for pathways involved in lymphocyte post-transcriptional regulation and cytokine–receptor interactions, while the high-risk group predominantly exhibited enrichment of cell cycle–related signaling pathways (Fig. 6C–F).

### 3.8 Assessment of Model Performance across Clinical Parameters

**Figure 7:**
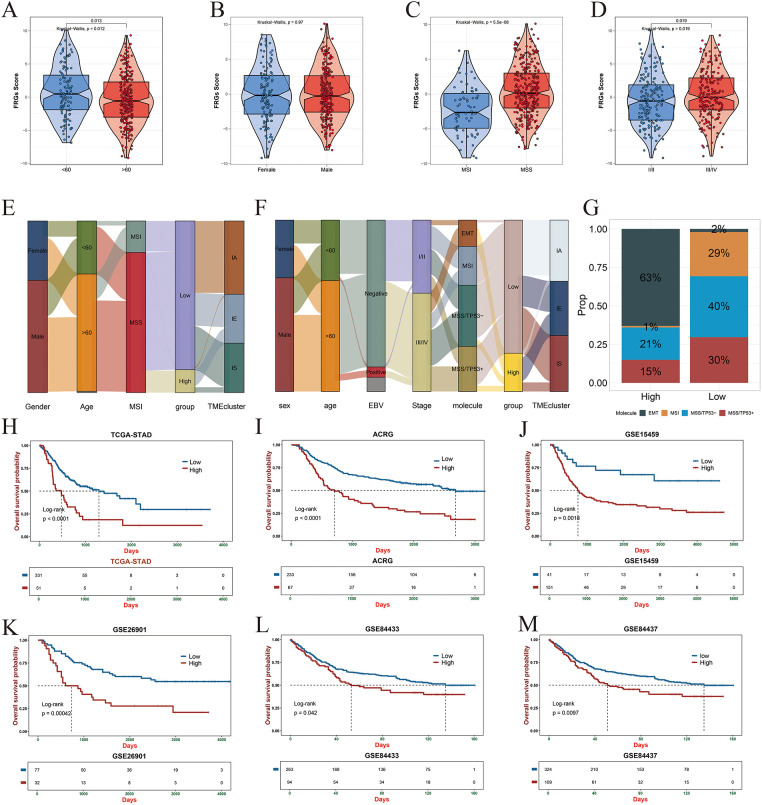
Association of FRG-based risk stratification with clinical and pathological features. (**A**) Comparison of FRG scores across different age groups. (**B**) Differences in FRG scores between male and female patients. (**C**) Analysis of FRG score distribution among microsatellite instability (MSI) subtypes (MSS, MSI-L, MSI-H). (**D**) FRG score differences across clinical staging groups (I–IV). (**E**,**F**) Sankey diagrams illustrating the relationship between FRG risk classification and clinical features in the TCGA (training set) and ACRG (validation set) cohorts. (**G**) Distribution of gastric cancer molecular subtypes across high- and low-risk groups in the ACRG cohort. (**H**–**M**) Kaplan–Meier survival analyses comparing overall survival between high- and low-risk groups in multiple gastric cancer cohorts, including TCGA (**H**), ACRG (**I**), GSE15459 (**J**), GSE26901 (**K**), GSE84433 (**L**), and GSE84437 (**M**).

In the training cohort, the fibroblast-related gene (FRG) risk score showed significant associations with key clinical features, including age, microsatellite stability/microsatellite instability (MSS/MSI) status, and tumor stage. Patients classified as high-risk were more likely to be younger, belong to the MSS subtype, and present with advanced-stage disease (Fig. 7A–D). These associations were further visualized using a Sankey diagram, which illustrated the distribution of FRG risk groups across clinical characteristics (Fig. 7E). Similar trends were observed in the ACRG validation cohort, where high-risk patients exhibited more aggressive clinicopathological features (Fig. 7F). In contrast, the low-risk group displayed a higher proportion of MSI cases, suggesting potentially greater responsiveness to immunotherapy and other therapeutic interventions (Fig. 7G). Kaplan–Meier survival analyses across both the training and validation cohorts consistently demonstrated significantly poorer outcomes among high-risk patients (Fig. 7H–M).

### 3.9 Genomic Alteration Profiles between Risk Groups

**Figure A5:**
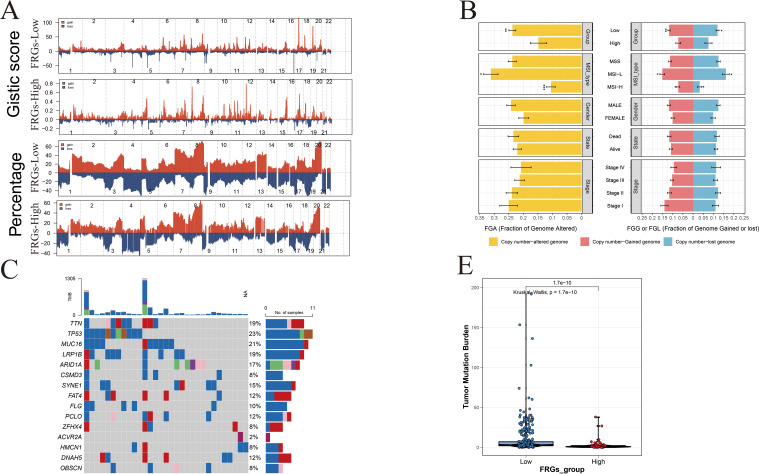

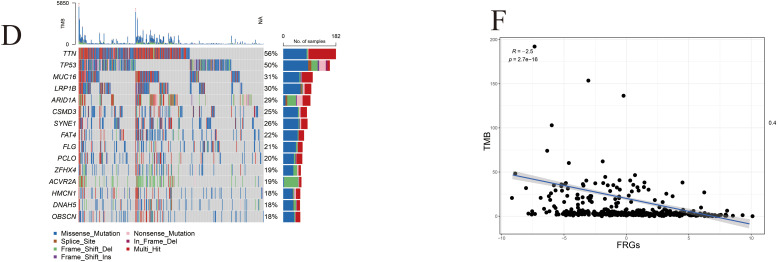
Genomic alteration landscape associated with FRG risk score in gastric cancer. (**A**) Comparative analysis of somatic mutation profiles between high- and low-risk groups in the TCGA-STAD cohort. (**B**) Differential assessment of gene alterations, including copy number gains and losses, stratified by FRG risk group, pathological subtype, and AJCC stage. (**C**,**D**) Frequency comparison of the top 15 commonly mutated FRG-related genes between high- and low-risk groups. (**E**) Analysis of mutation co-occurrence and exclusivity patterns in the two risk groups. (**F**) Correlation between the FRG risk score and tumor mutation burden (TMB), highlighting the relationship between genomic instability and risk stratification. *p* < 0.05 (*), *p* < 0.01 (**), *p* < 0.001 (***).

Somatic mutation analysis based on fibroblast-related gene (FRG) classification revealed that the low-risk group exhibited significantly higher GISTIC scores and increased frequencies of copy number variations (CNVs) within the TCGA gastric cancer cohort (Fig. A5A). Further assessment of genomic instability metrics—including the fraction of genome altered (FGA), fraction of genome gained (FGG), and fraction of genome lost (FGL)—demonstrated an elevated CNV burden in both the low-risk and MSI-L subgroups (Fig. A5B). These results indicate a positive association between FRG-based risk stratification and genomic instability. Notably, waterfall plots revealed a substantially higher frequency of TP53 mutations in the low-risk group (Fig. A5C,D), consistent with the observed increase in tumor mutational burden (TMB) within this subgroup (Fig. A5E,F).

### 3.10 FRG-Based Immune Landscape and Immunotherapy Response Prediction

**Figure 8:**
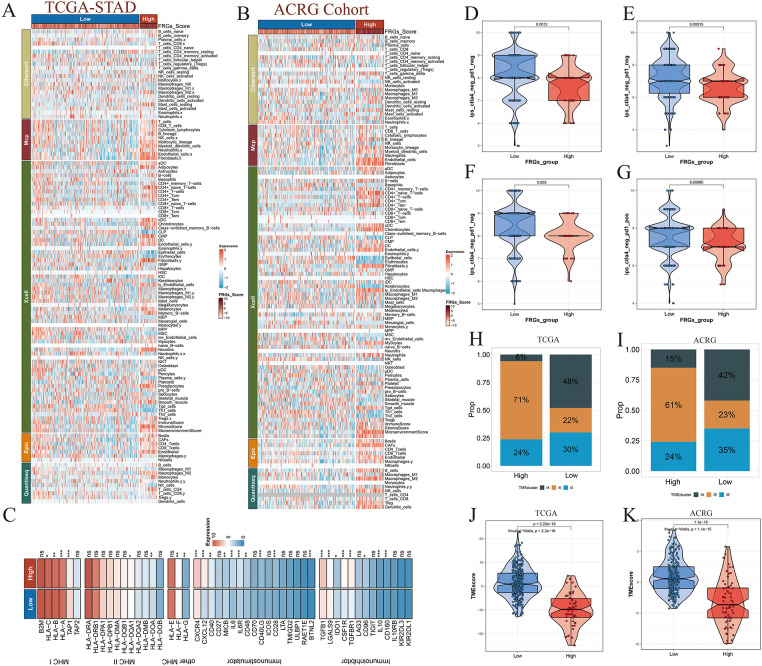
Immune profiling and checkpoint therapy prediction across risk groups. (**A**) Immune cell infiltration abundance in high- and low-risk groups within the TCGA-STAD cohort. (**B**) Immune cell infiltration patterns stratified by risk in the ACRG cohort. (**C**) Differential expression analysis of immune-related cytokines, chemokines, and immune checkpoint molecules between the two risk groups. (**D**–**G**) Immunophenoscore (IPS)-based prediction of immunotherapy efficacy for PD-1 and CTLA-4 blockade in high- and low-risk groups, highlighting differential immune responsiveness. (**H**,**I**) Distribution of immunophenotypes across risk groups, indicating variations in immune activation status. (**J**,**K**) Comparison of Tumor Microenvironment (TMEscore) values between high- and low-risk groups, reflecting immune and stromal enrichment. Significance levels were defined as follows: *p* < 0.05 (*), *p* < 0.01 (**), *p* < 0.001 (***), and *p* ≥ 0.05 (ns).

Immune infiltration patterns were largely consistent between the TCGA and ACRG cohorts, underscoring the robustness of immune landscape classification across independent datasets (Fig. 8A,B). Differential expression analysis of inflammatory cytokines revealed significant upregulation of immunosuppressive mediators in the high-risk group, indicative of a more immune-evasive tumor microenvironment (Fig. 8C). In contrast, patients classified as low-risk exhibited elevated immunophenoscores (IPS), suggesting increased predicted sensitivity to immune checkpoint blockade therapies targeting PD-1 and CTLA-4 (Fig. 8D–G). Furthermore, immune-activated molecular subtypes were more frequently observed among low-risk patients across both cohorts, reinforcing the immunologically favorable phenotype of this subgroup (Fig. 8H,I). Consistently, the tumor microenvironment score (TMEscore) was significantly higher in the low-risk group, reflecting enhanced activation of both immune and stromal components within the tumor microenvironment (Fig. 8J,K).

### 3.11 Aberrant Overexpression of TPP1 in Gastric Cancer

**Figure 9:**
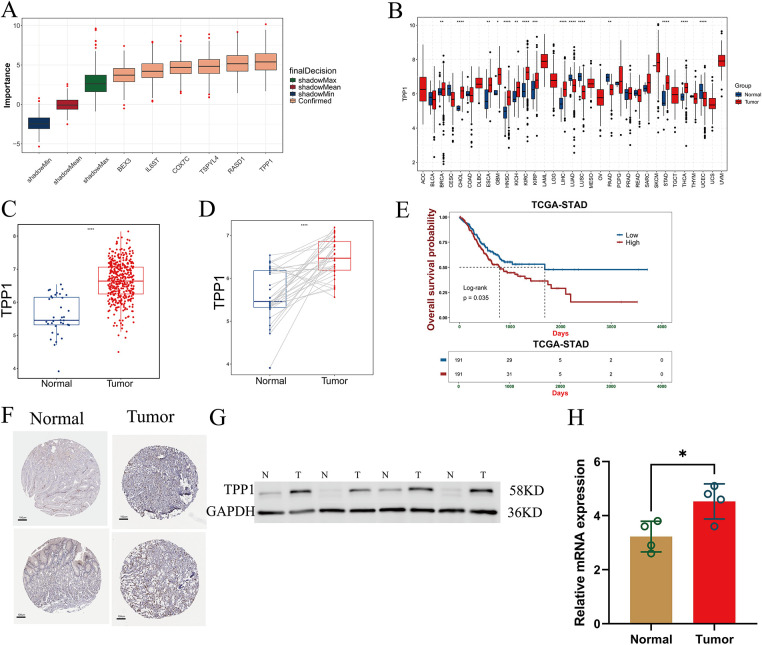
Abnormal expression of TPP1 was verified. (**A**) Boruta algorithm identified six key prognostic signature genes, with **TPP1** ranking as the top contributor. (**B**) Pan-cancer differential expression analysis revealed significant overexpression of **TPP1** across multiple malignancies. (**C**,**D**) Bar plots show that **TPP1** expression is significantly elevated in gastric cancer tissues based on both unpaired and paired comparisons. (**E**) Kaplan–Meier survival analysis demonstrated that high **TPP1** expression correlates with poorer overall survival in gastric cancer patients. (**F**) Immunohistochemical staining confirmed upregulated **TPP1** protein expression in gastric cancer tissues (HPA: The Human Protein Atlas). (**G**) Western blot analysis further validated increased **TPP1** protein levels in tumor samples. (**H**) qRT-PCR analysis confirmed that **TPP1** mRNA expression was significantly elevated in gastric cancer tissues (n = 4). Data are presented as mean ± standard deviation (SD). Statistical comparisons were performed using Student’s *t*-test unless otherwise indicated. Significance levels were defined as follows: *p* < 0.05 (*), *p* < 0.01 (**), *p* < 0.001 (***), and *p* < 0.0001 (****).

Boruta feature selection analysis identified six key genes from the 33 components of the prognostic model, among which TPP1 emerged as the most influential contributor (Fig. 9A). Pan-cancer differential expression analysis demonstrated that TPP1 was significantly overexpressed across a wide range of malignancies, including gastric cancer (Fig. 9B). Consistently, analysis of TCGA-STAD data confirmed marked TPP1 upregulation in gastric cancer tissues, as evidenced by both unpaired and paired differential expression analyses (Fig. 9C,D). Kaplan–Meier survival analysis further revealed that elevated TPP1 expression was significantly associated with poorer overall survival in patients with gastric cancer (Fig. 9E). These findings were corroborated by immunohistochemical staining, which showed increased TPP1 protein abundance in tumor tissues compared with adjacent normal counterparts (Fig. 9F). Moreover, qRT-PCR analysis of frozen clinical samples validated the robust overexpression of TPP1 in gastric cancer tissues (Fig. 9G,H).

### 3.12 Functional Validation of TPP1 as a Gastric Cancer Therapeutic Target

**Figure 10:**
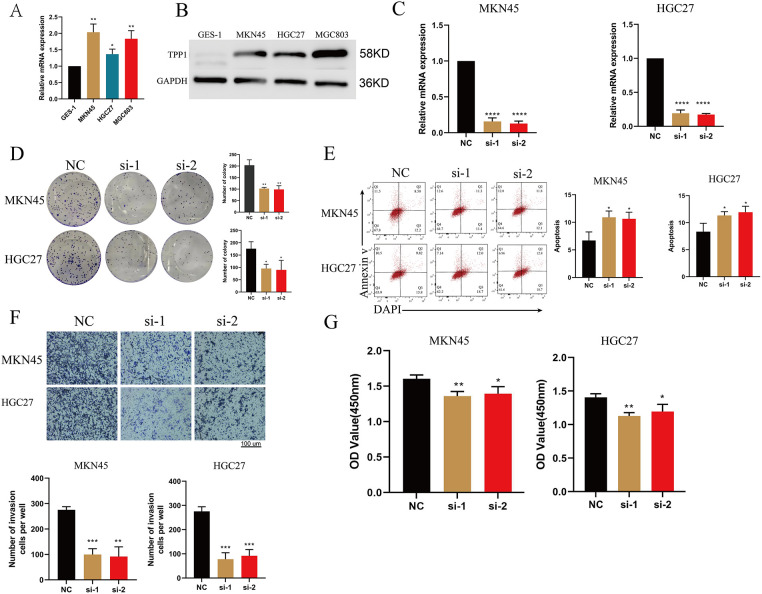
Silencing of TPP1 attenuates tumorigenic properties and enhances apoptosis in gastric cancer cells. (**A**) qRT-PCR analysis confirmed elevated TPP1 expression in gastric cancer cell lines (n = 3). (**B**) Western blot analysis validated high TPP1 protein levels across gastric cancer cell lines (n = 3). (**C**) qRT-PCR confirmed effective knockdown of TPP1 following siRNA transfection (n = 3). (**D**) Colony formation assays demonstrated a significant reduction in clonogenic capacity upon TPP1 silencing (n = 3). (**E**) Flow cytometry analysis using Annexin V/DAPI staining revealed increased apoptosis in TPP1-depleted cells (n = 3). (**F**) Transwell invasion assays showed a marked decrease in invasive capacity following TPP1 knockdown (n = 3). (**G**) CCK-8 assays indicated impaired cell viability in TPP1-silenced gastric cancer cells (n = 3). Data are presented as mean ± standard deviation (SD), and all experiments were performed in triplicate. Statistical significance was assessed using Student’s t-test unless otherwise specified: *p* < 0.05 (*), *p* < 0.01 (**), *p* < 0.001 (***), *p* < 0.0001 (****).

Quantitative PCR analysis revealed that TPP1 expression was significantly upregulated in gastric cancer cell lines compared with normal gastric epithelial controls (Fig. 10A). To elucidate its functional role in tumor progression, TPP1 was silenced using siRNA, and knockdown efficiency was confirmed at both the mRNA and protein levels by qRT-PCR and Western blotting, respectively (Fig. 10B,C). Functional assays demonstrated that TPP1 knockdown markedly reduced the clonogenic capacity of gastric cancer cells (Fig. 10D) and significantly increased apoptotic cell populations (Fig. 10E). Moreover, TPP1 suppression substantially impaired invasive potential (Fig. 10F) and diminished cell viability (Fig. 10G). Collectively, these findings underscore the oncogenic role of TPP1 in gastric cancer progression and highlight its promise as a potential therapeutic target.

## 4 Discussion

Gastric cancer remains one of the leading causes of cancer-related mortality worldwide and is characterized by pronounced heterogeneity that profoundly influences prognosis and therapeutic response [2,40]. Despite advances in surgical techniques and chemotherapeutic regimens, clinical outcomes remain unsatisfactory, largely due to high recurrence rates and metastatic dissemination, particularly peritoneal spread [41,42]. This underscores the urgent need to identify novel therapeutic targets that can enhance treatment efficacy and ultimately improve clinical outcomes for patients with gastric cancer.

Cancer-associated fibroblasts (CAFs) play pivotal roles in various aspects of tumor progression. By secreting growth factors, pro-inflammatory cytokines, and extracellular matrix (ECM) components, CAFs promote cancer cell proliferation, therapeutic resistance, and immune evasion [13]. In addition, emerging evidence highlights the critical role of cancer-associated fibroblast (CAF) heterogeneity in shaping the immunosuppressive tumor microenvironment [43]. Beyond immune regulation, CAFs are also implicated in promoting tumor progression, metabolic reprogramming, and therapeutic resistance [44]. In this study, we identified eight metastasis-associated fibroblast subpopulations in gastric cancer, with tissue preference analysis revealing specific CAF subtypes that drive metastatic progression. The gene signatures of these subpopulations were significantly associated with poor prognosis, underscoring their prognostic relevance. Transcriptomic analyses further demonstrated that metastasis-linked markers—APOE, CLDN1, and DLK1—were strongly correlated with unfavorable clinical outcomes, highlighting their potential as indicators of aggressive gastric cancer phenotypes. Notably, APOE has been implicated in promoting tumor invasion and epithelial–mesenchymal transition (EMT) [45], while CLDN1 has been shown to enhance tumor stemness and resistance to therapy [46].

Machine learning algorithms, renowned for their powerful data processing and pattern recognition capabilities, have shown substantial potential in integrating large-scale omics datasets and constructing predictive models [47–49]. Among these approaches, the Least Absolute Shrinkage and Selection Operator (LASSO) regression has demonstrated exceptional effectiveness in identifying diagnostic biomarkers and prognostic indicators across diverse malignancies [33,50,51]. LASSO is particularly effective for feature selection in high-dimensional datasets, facilitating efficient model optimization and enhancing interpretability. Furthermore, the integration of LASSO with ensemble learning approaches, such as the random forest algorithm, has gained increasing prominence in recent years, especially in the identification of therapeutic targets and the development of prognostic models in oncology [52,53]. This combinatorial approach enhances predictive accuracy and facilitates individualized risk stratification. In the present study, we applied both LASSO regression and random forest algorithms to construct a prognostic model based on metastasis-associated fibroblast (MAF) signature genes in gastric cancer. The resulting model exhibited robust prognostic performance, effectively stratifying patients according to survival outcomes. Moreover, its predictive capacity extended to associations with clinical characteristics, immune cell infiltration profiles, and immunotherapeutic responsiveness, highlighting its potential to guide precision treatment strategies for patients with gastric cancer.

In this study, the prognostic model based on metastasis-associated fibroblasts (MAFs) effectively stratified gastric cancer patients according to their immune background and mutational profiles in an independent validation cohort. High-risk patients exhibited an immunosuppressive tumor microenvironment, characterized by reduced immune cell infiltration, diminished expression of antitumor immune effectors, and poor responsiveness to immunotherapy, all of which were closely associated with unfavorable prognosis. The strong association between the proposed prognostic model and the immune landscape further underscores the pivotal role of the tumor microenvironment in shaping therapeutic outcomes, particularly in the context of immunotherapy. Previous studies have demonstrated that dynamic interactions among immune cells, stromal cells, inflammatory mediators, and tumor-derived factors within the tumor microenvironment can either promote antitumor immunity or facilitate immune evasion. Moreover, the functional states and spatial organization of T cells, tumor-associated macrophages, and tumor-associated fibroblasts have been shown to critically influence immunotherapeutic efficacy [10,54]. In addition, cytokines such as TGF-β and IL-10 can lead to drug resistance by recruiting immune cells and promoting polarization [55,56]. These findings further reveal the importance of tumor microenvironment heterogeneity in risk stratification and prognosis of gastric cancer and prediction of immunotherapy efficacy.

TPP1, a telomerase end-binding protein, has been shown to facilitate telomere elongation in tumor cells, thereby promoting oncogenesis [57]. In addition to its telomeric function, TPP1^+^ macrophages have been reported to enhance tumor progression by inducing methylation of p53 [58]. In the context of immunotherapy, peptide-mediated blockade of TPP1 has been shown to markedly enhance therapeutic efficacy in lung cancer models [59]. Moreover, TPP1 has been implicated in the suppression of DNA damage responses and the reduction of chemotherapy sensitivity in tumor cells, further underscoring its multifaceted role in promoting therapeutic resistance [60]. *In vitro* experiments confirmed a marked upregulation of TPP1 in gastric cancer tissues. Knockdown of TPP1 significantly suppressed cellular invasion, clonogenicity, and viability while promoting apoptosis, thereby confirming its oncogenic function and therapeutic potential in gastric cancer.

Despite the comprehensive design and multi-level validation of this study, several limitations should be acknowledged. First, the analyses primarily relied on publicly available datasets (TCGA and GEO), and the retrospective nature of these data may introduce potential selection bias and limit generalizability. Second, although single-cell transcriptomic data enabled detailed characterization of fibroblast heterogeneity, the sample size of the single-cell cohort was relatively limited, which may not fully capture interpatient variability across different gastric cancer subtypes and clinical stages. Third, while the prognostic model was validated across multiple independent cohorts, further prospective clinical studies with larger and more diverse populations are required to confirm its predictive robustness and clinical utility. Fourth, the functional validation of the core gene TPP1 was restricted to *in vitro* assays; additional *in vivo* experiments and mechanistic studies are needed to elucidate the precise molecular pathways through which TPP1 and other fibroblast-associated genes influence tumor progression and immune modulation. Finally, potential batch effects and technical heterogeneity inherent to multi-omics data integration may have influenced certain analyses despite rigorous normalization and correction procedures.

## 5 Conclusion

In summary, this study delineated the heterogeneity of fibroblasts within the gastric cancer microenvironment through single-cell transcriptomic analysis and developed a robust prognostic model based on metastasis-associated fibroblast signatures by integrating multi-omics datasets. The model demonstrated strong efficacy in stratifying patients according to clinical prognosis, immune landscape, immunotherapeutic responsiveness, and genomic alterations. These findings provide a compelling framework for advancing precision oncology and individualized patient stratification in gastric cancer.

## Data Availability

The original contributions of this study are included in the article or Appendix B. Please contact the corresponding author for further data or code.

## Appendix B

The results of mycoplasma detection of gastric cancer cell lines showed that the gastric cancer cell lines (MKN45, HGC27, MGC803) used in this study were free of mycoplasma contamination. Mycoplasma primer synthesis was completed by Sangon. RNA extraction kit was from Lucigen (QE0905T). Sample wells are labeled MKN45, HGC27, MGC803 and negative control.

The primers used for the detection are as follows:

**Table:**

Primer Name	Primer Sequence (5^′^-3^′^)
Myco-F-1	cgc ctg agt agt acg twc gc
Myco-F-2	tgc ctg rgt agt aca ttc gc
Myco-F-3	cgc ctg agt agt atg ctc gc
Myco-F-4	cgc ctg ggt agt aca ttc gc
Myco-R-1	gcg gtg tgt aca ara ccc ga
Myco-R-2	gcg gtg tgt aca aac ccc ga
